# Large Tuberculous Mass Lesions Involving the Brain: Outcomes and Management

**DOI:** 10.4269/ajtmh.24-0376

**Published:** 2025-04-01

**Authors:** Prasannakumar Palanikumar, Priyanka Gautam, Harshad Arvind Vanjare, Nagaraj Veerasamy, Mithun Mohan George, Leeberk Raja Inbaraj, Edmond Jonathan Gandham, Ajith Sivadasan, Rajiv Karthik, Abi Manesh

**Affiliations:** ^1^ Department of Infectious Diseases, Christian Medical College, Vellore, India;; ^2^ Department of Radiology, Christian Medical College, Vellore, India;; ^3^ Department of Clinical Research, Indian Council of Medical Research-National Institute for Research in Tuberculosis, Chennai, India;; ^4^ Division of Neurosurgery, Christian Medical College, Vellore, India;; ^5^ Division of Neurology, Department of Neurological Sciences, Christian Medical College, Vellore, India

## Abstract

The optimal management of large tuberculous mass lesions (LTML) involving the central nervous system remains unclear. We conducted a single-center, retrospective, observational study that assessed the outcomes of patients with LTML from January 2010 to February 2023. An LTML was defined as a tuberculoma or tubercular abscess exceeding or equal to 3 cm. The primary outcome was independence in activities of daily living, as assessed by the modified Rankin Scale (mRS) at a follow-up of 6 months. Forty-six patients were identified during the study period. Their mean age was 27.6 ± 12 years, the median duration of antituberculous therapy (ATT) was 18 months, and the median duration of follow-up was 20 months (interquartile range 15.7–40). The favorable outcomes were 76.9% (10/13) for ATT alone, 62.5% (10/16) for ATT with steroids, 87.5% (7/8) for ATT with surgery, and 66.9% (6/9) for ATT, steroids, and surgery. The median mRS at baseline in the study was 2 (1–3), and at the 6 month follow-up, it was 1 (0–2). Adding steroids or surgical intervention to ATT did not significantly improve primary outcomes (*P* = 0.637). Further large-scale studies are necessary to confirm these preliminary observations.

## INTRODUCTION

Central nervous system (CNS) tuberculosis (TB) is a severely debilitating and potentially life-threatening illness.^1^ Although tubercular meningitis has been extensively studied, there is limited research on the management and outcomes of tubercular mass lesions, particularly large tuberculous mass lesions (LTML). These lesions often cause significant mass effects and present unique treatment challenges. The optimal management approach for LTML remains controversial, with some clinicians advocating for upfront surgical intervention, whereas others prefer conservative management with antitubercular therapy and steroids.^2^ Our study aims to bridge this knowledge gap.

## MATERIALS AND METHODS

### Study design.

We conducted a single-center, retrospective observational study of patients with CNS LTML from January 2010 to February 2023 at a 3,800-bed quaternary care center in South India.

### Participants.

Adult patients (aged ≥15 years) with intracranial mass lesions sized ≥3 cm and radiological evidence suggestive of tuberculoma (brain magnetic resonance imaging: well-marginated enhancing lesions with or without meningitis; brain computed tomography (CT): peripherally enhancing lesion with a low attenuation center surrounded by vasogenic edema) were included in the study. Patients with confirmed alternative etiologies were excluded. All patients received antitubercular therapy (ATT), with or without steroids and surgery, as treatment.

### Data collection and follow-up.

We documented patients’ demographics, neurological signs and symptoms, treatment, and both baseline and follow-up imaging. We recorded the clinical and neurological outcomes of the patients during the baseline and follow-up visits at 6 months using a modified Rankin score (mRS). Telephone calls were made when follow-up data were incomplete.

### Primary outcome.

The primary outcome was independence in activities of daily life (scores of 0, 1, and 2) at 6 months, as assessed by the mRS, compared with baseline, or the resolution of all symptoms or signs if the patient was already independent at baseline.

## STATISTICAL ANALYSES

The descriptive statistics were analyzed using frequency and percentages for categorical variables, as well as mean and SD and median and interquartile range (IQR), for continuous variables. Associations between good outcomes and clinical predictors were assessed using the appropriate χ^2^ or Fisher’s exact test. Continuous variables were compared using the independent *t*-test or Mann–Whitney *U* test, depending on the distribution. The Wilcoxon signed rank test was used to assess the median difference between absolute baseline and follow-up mRS scores. Factors with a *P* <0.05 were included in the multivariate analysis. The independent effects of factors associated with good outcomes were evaluated using multiple logistic regression. All analyses were performed using the statistical software IBM SPSS Statistics (Version 23; IBM Corp., Armonk, NY).

## RESULTS

We screened patients using our picture archiving and communication system for magnetic resonance/CT imaging, identifying any CNS mass lesions from 2010. Of the total 1,641 patients with ring-enhancing lesions in the brain, 169 had lesions measuring greater than or equal to 3 cm with various etiologies, including tuberculomas, gliomas, neurocysticercosis, and pyogenic abscesses. Among these, 46 patients with tuberculous LTML were included in the study.

The mean age of the patients was 27.6 years (SD ± 12), and the majority were female (25/46; 54.3%). None of the patients had concomitant HIV infection. Twenty-two (48%) patients had microbiologically confirmed TB, of which most cases were rifampicin-susceptible TB (44/46; 95.7%), and 52% had a biopsy showing granulomatous inflammation. Two patients had multidrug-resistant TB. Sixteen patients (34.8%) had probable tuberculomas based on clinical and radiological imaging findings alone. Almost half (24/46; 52.2%) had disseminated TB, and one-quarter (11/44; 25%) had chest X-rays suggestive of TB (as detailed in Supplemental Table 1). The median duration of antituberculous therapy (ATT) was 18 months (IQR 15–24), and the median duration of follow-up was 20 months (IQR 15.5–40). Two patients were reported dead at follow-up.

The index clinical presentations were focal neurological deficit (24/46, 52.2%), seizure (22/46, 47.8%), and symptoms of raised intra cranial pressure (ICP) (11/46, 23.9%). The median duration of illness at presentation was 4 months (IQR 2–9). On imaging, 30 of 46 (65.2%) patients had a midline shift, and 6 of 46 (13%) had lesions measuring greater than 5 cm. Almost one-third of patients (14/46 [30.4%]) had tuberculomas and tuberculous meningitis. A third of patients (14/46 [30.4%]) had lesions in the frontal lobe, followed by the cerebellar (9/46 [19.6%]), parietal (8/46 [17.4%]), and temporal (6/46 [13%]) regions; 6 of 46 (13%) patients had extensive lesions involving multiple lobes, and 3 of 46 (6.5%) had thalamic lesions.

The primary outcome was observed in 33 of 46 patients (71.7%). The median absolute mRS at baseline in the study was 2 (1–3), and at the 6-month follow-up, it was 1 (0–2). In Supplemental Table 2, only focal neurological deficits (RR 0.2 [95% CI 0.05–0.95]; *P* = 0.043) showed a significant risk for poor outcomes in univariate analysis. However, when analyzed in a multivariate model, none were found to be statistically significant.

Radiologically, the majority of patients (27/41 [65.8%]) showed significant improvement, 10 of 41 (24.3%) showed moderate improvement, and 4 of 41 (9.7%) showed no improvement. Additionally, 5 of 46 (10.8%) did not undergo follow-up imaging (Supplemental Figure 1). All patients received one of the following four possible therapy modalities: 13 of 46 (28.3%) received ATT alone, 16 of 46 (34.8%) received ATT and steroids, 8 of 46 (17.4%) received ATT with tuberculoma excision, and 9 of 46 (19.6%) received ATT, steroids, and tuberculoma excision. A significant number of patients (17/46 [37%]) underwent surgery. Better outcomes were not noted with surgery (87.5%) or the addition of steroids (62.5%) to antimycobacterial therapy (*P* = 0.637). In the subgroup analysis of 34 of 46 (73.9%) patients with signs and symptoms of raised ICP with midline shift, no difference in successful outcomes was observed between patients who underwent surgery and those managed conservatively with only medical therapy (10/13 [76.9%]) versus 3/13 [23.1%] and 15/21 [71.4%] versus 6/21 [28.6%]; *P* = 1.00).

## DISCUSSION

To the best of our knowledge, this study represents the first comprehensive evaluation of patients’ presentations, treatment strategies, and outcomes associated with LTML. Despite the large lesions and associated edema, most patients had good outcomes at 6 months. Our outcomes are superior to those of many CNS tuberculoma cohorts across the globe and similar to other reports from this region.^3^^–^^6^ Low rates of HIV co-infection, drug-resistant TB, and secular trends in management strategies over the years may explain these findings.

An important question in the management of LTML concerns the role of steroids and surgery as adjuncts to antitubercular therapy. Although specific data concerning LTML are not available, some guidelines endorse the use of steroids in the management of CNS TB beyond TB meningitis.^7^ However, a clinical trial or well-matched prospective study has yet to evaluate this question.

Steroid use remains prevalent in tuberculoma cohorts.^3^^,^^4^^,^^6^ Nevertheless, steroids may impair the penetration of antitubercular agents, particularly isoniazid, into the CNS, and tuberculomas often develop in TB meningitis during treatment despite corticosteroid therapy.^8^^,^^9^ In our study, steroid administration was not associated with improved outcomes. Furthermore, steroids do not appear to influence the development or resolution of CNS mass lesions, especially when the size exceeds 3 cm.^3^

A small subset of patients with LTML who experience severe paradoxical worsening, life-threatening mass effects, or the involvement of specific locations, such as the posterior fossa, may benefit from steroids and surgery.^10^^,^^11^ However, the routine use of steroids and surgical intervention in LTML may not be warranted and requires further investigation.

Our study has several limitations. The retrospective, observational nature and limited sample size present challenges in evaluating therapeutic interventions. Additionally, not all patients had microbiologically confirmed TB. In conclusion, our preliminary data indicate that the majority of patients with LTML demonstrate favorable neurological outcomes at 6 months, and the routine use of high-dose steroids and surgery necessitates further study.

## Supplemental Materials

10.4269/ajtmh.24-0376 Supplemental Materials

## References

[1] Navarro-Flores A Fernandez-Chinguel JE Pacheco-Barrios N Soriano-Moreno DR Pacheco-Barrios K, 2022. Global morbidity and mortality of central nervous system tuberculosis: A systematic review and meta-analysis. J Neurol 269: 3482–3494. 35288778 10.1007/s00415-022-11052-8 PMC8920747

[2] Marais S Van Toorn R Chow FC Manesh A Siddiqi OK Figaji A Schoeman JF Meintjes G; Tuberculous Meningitis International Research Consortium, 2019. Management of intracranial tuberculous mass lesions: How long should we treat for? Wellcome Open Res 4: 158. 32047859 10.12688/wellcomeopenres.15501.3 PMC6996525

[3] Marais S Roos I Mitha A Patel V Kalincik T Bhigjee AI, 2020. Presentation and outcome of patients with intracranial tuberculoma in a high HIV prevalence setting. Int J Tuberc Lung Dis 24: 224–232. 32127108 10.5588/ijtld.19.0386

[4] Wasay M Moolani MK, 2004. Prognostic indicators in patients with intracranial tuberculoma: A review of 102 cases [published correction appears in J Pak Med Assoc 54: 401. Smego, AR (corrected to Smego, RA)]. J Pak Med Assoc 54: 83–87. 15134209

[5] Harder E Al-Kawi MZ Carney P, 1983. Intracranial tuberculoma: Conservative management. Am J Med 74: 570–576. 6837585 10.1016/0002-9343(83)91011-2

[6] Rajeswari R Sivasubramanian S Balambal R Parthasarathy R Ranjani R Santha T Somasundaram PR Ganapathy S Sudarsana K Sayeed ZA, 1995. A controlled clinical trial of short-course chemotherapy for tuberculoma of the brain. Tuber Lung Dis 76: 311–317. 7579312 10.1016/s0962-8479(05)80029-2

[7] Thwaites G Fisher M Hemingway C Scott G Solomon T Innes J; British Infection Society, 2009. British Infection Society guidelines for the diagnosis and treatment of tuberculosis of the central nervous system in adults and children. J Infect 59: 167–187. 19643501 10.1016/j.jinf.2009.06.011

[8] Sarma GR Kailasam S Nair NG Narayana AS Tripathy SP, 1980. Effect of prednisolone and rifampin on isoniazid metabolism in slow and rapid inactivators of isoniazid. Antimicrob Agents Chemother 18: 661–666. 7447424 10.1128/aac.18.5.661 PMC284072

[9] Lu Y Hu Z Wang F Yao H Zhu H Wang Z Song Z Chen R Liu D, 2020. Worsening CSF parameters after the start of anti-tuberculosis treatment predicts intracerebral tuberculoma development. Int J Infect Dis 101: 395–402. 33002621 10.1016/j.ijid.2020.09.1457

[10] Rajshekhar V, 2015. Surgery for brain tuberculosis: A review. *Acta Neurochir (Wien)* 157: 1665–1678. 26170188 10.1007/s00701-015-2501-x

[11] Suárez I , 2020. Intensified adjunctive corticosteroid therapy for CNS tuberculomas. Infection 48: 289–293. 31900872 10.1007/s15010-019-01378-3

