# Supplementary material for: Large Tuberculous Mass Lesions Involving the Brain: Outcomes and Management
Source: Am J Trop Med Hyg. 2025 Apr 1;112(6):1264–6. doi: 10.4269/ajtmh.24-0376 (PMC12139545; doi:10.4269/ajtmh.24-0376)
Supplement: Supplemental Materials [file tpmd240376.SD1.pdf]

Supplemental Table 1
Baseline characteristics of patients with large tubercular mass lesions

**Variable**	**Total†** **(*N* = 46)**	**Good Outcome (*n* = 33)**	**Poor Outcome (*n* = 13)**	***P*-Value**
Age, Mean ± SD	27.6 ± 12	29.0 ± 13.3	24.3 ± 7.5	0.574
Sex, Male (%)	21 (45.7)	14 (66.7)	7 (33.3)	0.484
Biopsy proven (%), *n* = 24	24 (52.2)	20 (83.3)	4 (16.7)	-
Microbiologically confirmed TB* (%)	22 (47.8)	16 (72.7)	6 (27.3)	0.887
Disseminated TB (%)	24 (52.2)	18 (75)	6 (25)	0.608
Resistance (%)	2 (4.3)	1 (50)	1 (50)	0.490
Clinical Features				
Symptoms of raised ICP (%)	11 (23.9)	11 (100)	0	0.020
Seizure (%)	22 (47.8)	14 (63.6)	8 (36.4)	0.243
Focal neurological deficits (%)	24 (52.2)	14 (58.3)	10 (41.7)	0.035
Cerebellar signs (%)	9 (19.6)	7 (77.8)	2 (22.2)	1.000
Paradoxical worsening present (%)	4 (8.7)	3 (75)	1 (25)	1.000
Laboratory Parameters				
CRP >6 mg/L (%), *n* = 22	9/22 (40.9)	6/9 (66.7)	3/9 (33.3)	1.000

Abnormal CSF (%), *n* = 11	8/11 (72.7)	4/8 (50)	4/8 (50)	1.000
**Imaging**				
Lesion size, >5 cm (%)	6 (13)	4 (66.7)	2 (33.3)	1.000
Midline shift present (%)	30 (65.2)	21 (70)	9 (30)	1.000
Abnormal Chest X-Ray (%), *n* = 44	11/44 (25)	7/11 (63.6)	4/11 (36.4)	0.457
**Treatment**				
Received ATT alone (%)	13 (28.3)	10 (76.9)	3 (23.1)	0.637
Received ATT+ Steroids (%)	16 (34.8)	10 (62.5)	6 (37.5)	
Received ATT+ Surgery (%)	8 (17.4)	7 (87.5)	1 (12.5)	
Received ATT+ Steroids + Surgery (%)	9 (19.6)	6 (66.7)	3 (33.3)	
Received Non-steroid Immunomodulators (%)	5 (10.9)	4 (80)	1 (20)	1.000
Underwent Surgery (%)	17 (37)	13 (76.7)	4 (23.5)	0.739
On medical management alone (%)	29 (63)	20 (69)	9 (31)	

***** Microbiologically confirmed TB **-** comprises of either Xpert or Culture positive patients
[†]First column 'Total' presents the column percentages; while other percentages are row percentages.

Supplemental Table 2
Multivariate logistic regression analysis for predictors of good outcome among patients with large tubercular mass lesions

**Variable**	**Good Outcome (*n* = 33)**	**Poor Outcome (*n* = 13)**	**RR (95% CI)**	***P*-Value**	**aRR (95% CI)**	***P*-Value**
Microbiologically confirmed (%)	16 (72.7)	6 (27.3)	0.9 (0.26-3.52)	0.966	1.6 (0.33-7.78)	0.544
Focal neurological deficits (%)	14 (58.3)	10 (41.7)	0.2 (0.05-0.95)	0.043	0.2 (0.04-1.09)	0.065
Midline shift present (%)	21 (70)	9 (30)	0.7 (0.19-3.07)	0.720	0.7 (0.16-3.17)	0.657
Received ATT alone (%)	10 (76.9)	3 (23.1)	Reference	-	Reference	-
Received ATT+ Steroids (%)	10 (62.5)	6 (37.3)	0.5 (0.09-2.57)	0.407	0.5 (0.10-3.26)	0.538
Received ATT+ Surgery (%)	7 (87.5)	1 (12.5)	2.1(0.17-24.59)	0.555	1.5(0.11-20.89)	0.753
Received ATT+ Steroids + Surgery (%)	6 (66.7)	3 (33.3)	0.6 (0.09-3.98)	0.597	0.6 (0.07-5.16)	0.664

Supplemental Figure 1

Patient 1

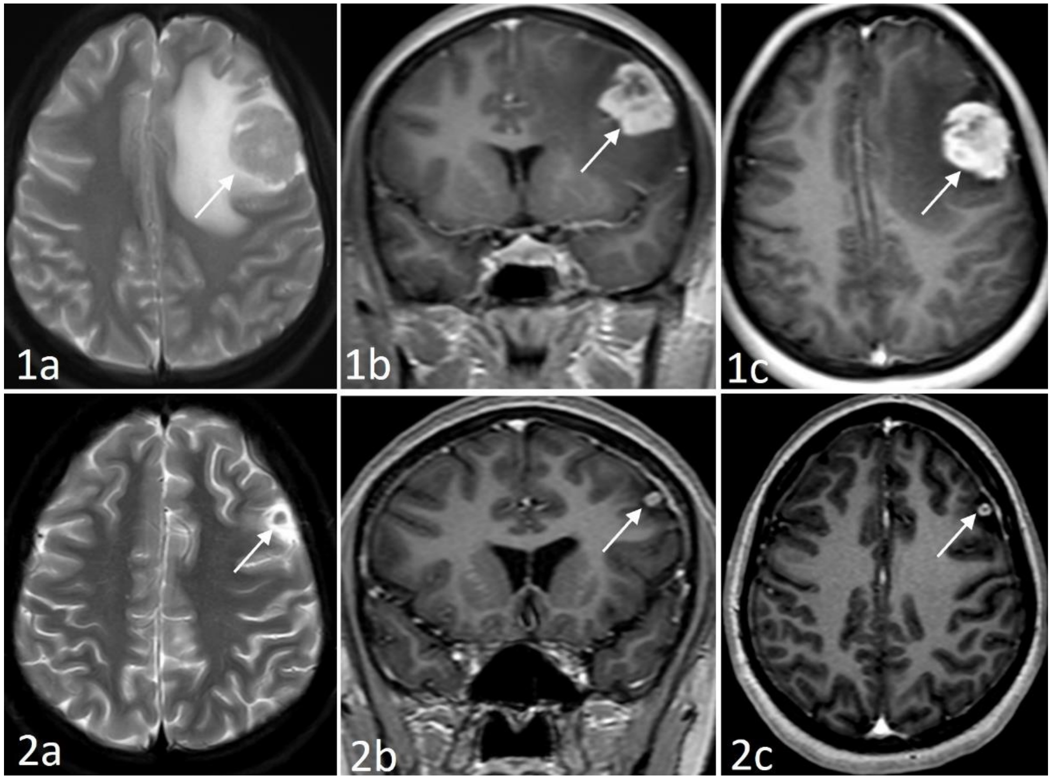

Patient 2

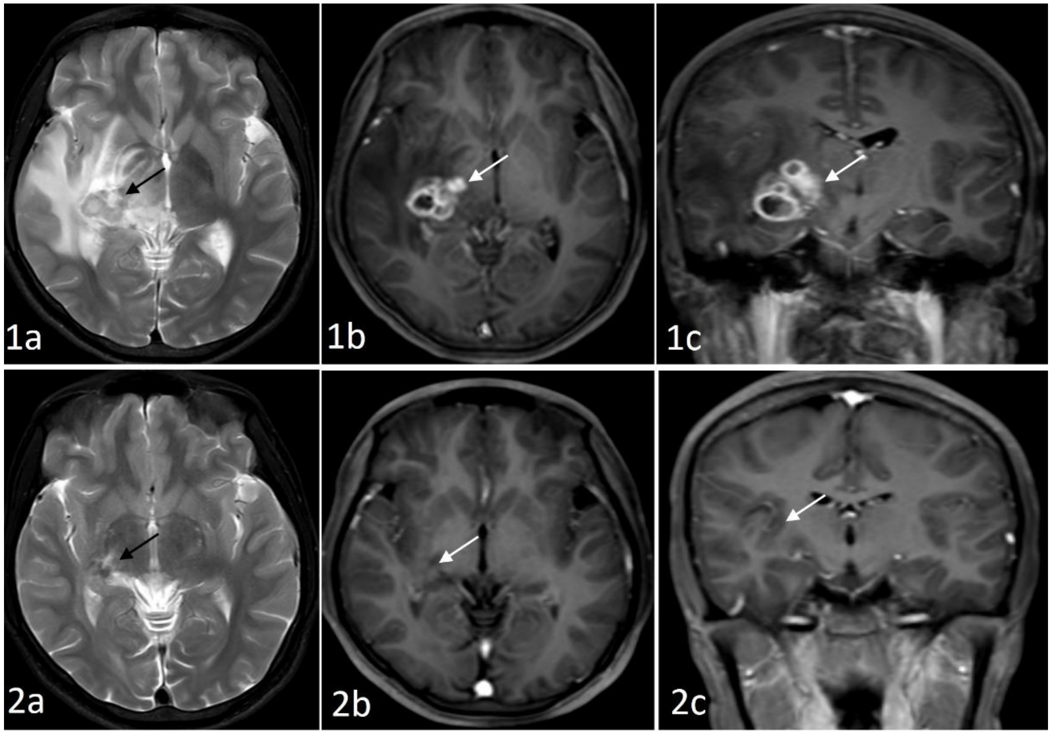

Supplemental Figure 1. Pre and post treatment images of two patients with large tuberculous mass lesions.
Patient 1: 16-year-old female with seizures. MR imaging showed a mass-like lesion involving the left frontal lobe with significant surrounding oedema on T2W **(1a)**. This lesion showed intense enhancement in the post-contrast study **(1b and 1c)**. Follow-up imaging of 5 months of anti-tuberculous therapy showed significant regression of this lesion **(2a)** and post-contrast enhancement **(2b and 2c).** Patient 2: 18-year-old male with left-sided deficits. MR imaging showed a heterogeneous lesion involving the posterior aspect of the right lentiform nucleus with significant surrounding oedema on T2W **(1a)**. This lesion appeared as a conglomerate ring-enhancing lesion in the post-contrast study **(1b and 1c)**. Follow-up imaging at 2.5 years following anti-tuberculous therapy showed significant regression of this lesion **(2a)** and near total resolution of post-contrast enhancement **(2b and 2c).**
